# Stepping back for good reasons: a reappraisal of the DF-1 connector for defibrillator leads

**DOI:** 10.1093/europace/euae057

**Published:** 2024-02-27

**Authors:** Christian Sticherling, Kenneth A Ellenbogen, Haran Burri

**Affiliations:** Department of Cardiology, University Hospital Basel, University of Basel, Petersgraben 4, CH-4059 Basel, Switzerland; Department of Electrophysiology, Virginia Commonwealth University, Richmond, VA, USA; Department of Cardiology, University Hospital Geneva, Geneva, Switzerland

**Keywords:** Implantable cardioverter defibrillator, Pacing therapy, Cardiac resynchronization therapy, Lead malfunction, Risk benefit, Device upgrade, Device downgrade, Conduction system pacing

## Abstract

The DF-4 defibrillator standard has been rapidly adopted due to its convenience at implantation. There are however trade-offs compared to the traditional DF-1 standard that are underappreciated. This viewpoint outlines the advantages and limitations of current defibrillator lead standards that should be kept in mind, as they impact the options that are available to deal with issues that may arise.

## Graphical Abstract

**Graphical Abstract:**
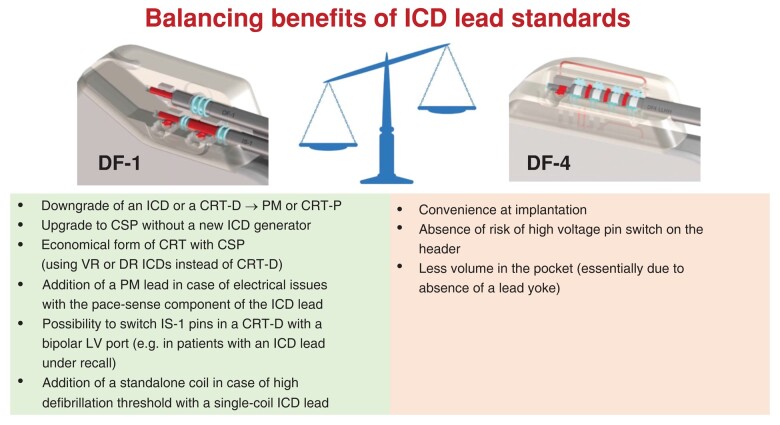
CRT, cardiac resynchronization therapy; CRT-D, cardiac resynchronization therapy implantable defibrillator; CRT-P, cardiac resynchronization therapy pacemaker; CSP, conduction system pacing; DR, dual-chamber; ICD, implantable cardioverter defibrillator; PM, pacemaker; VR, single-chamber.

**What’s new?**

- The DF-4 defibrillator standard has been rapidly adopted.
- The DF-1 defibrillator standard allows for more flexibility during follow-up (e.g. downgrading from an ICD to a pacemaker, upgrading to conduction system pacing without a new generator, and addition of PM leads).
- The DF-1 defibrillator standard should not be abandoned.

## Viewpoint

Pacemaker and implantable cardioverter defibrillator (ICD) leads are connected to the respective pulse generators by specific connectors. To allow the interchangeability between devices of different manufacturers, unified industry standards were introduced in the early 1990s. IS-1 denotes the standard for bipolar pacemaker leads while IS-4 applies to quadripolar left ventricular leads used for cardiac resynchronization therapy (CRT). Defibrillator leads are much more complex as they comprise connections for one or two high-voltage coils in addition to the low-voltage pace–sense component. This resulted in the original DF-1 ICD leads having a yoke with a bifurcation or trifurcation at the proximal end of the lead, and consequently larger device headers that had to hold three to five ports.

To overcome this problem, a single connection combining the low- and high-energy components of the ICD lead was introduced by Abbott in 2010—the DF-4 standard. Furthermore, DF-4 ICD leads completely avoid erroneous connection of the DF-1 plugs.

The advantages of this lead design are summarized in the Graphical Abstract.

### Differences in outcome in patients with DF-1 and DF-4 leads

Despite initial safety concerns because of the proximity of the low- and high-voltage components on a single pin,^1^ the DF-4 defibrillator connector has been proved to be safe and reliable over the past decade.^2^ Because of the ease of handling and less lead material in the pocket (related to the absence of a yoke, rather than to downsizing of the generator), DF-4 leads have become the preferred standard for most operators.

There are nevertheless limitations with the DF-4 standard, which are of concern and are outlined below.

### Clinical scenarios for the need for an additional ventricular pace/sense lead

#### Downgrade from defibrillator to pacemaker systems

During the course of time, patients may no longer need the defibrillator function of their CIED anymore. This may be the case for super-responders to CRT or in elderly patients requiring anti-bradycardia pacing and who do not wish defibrillator therapy at the time of generator change.^3,4^ This is pertinent since the defibrillator not only adds cost but is also bulkier than a pacemaker and increases the risk for infection with every change.

The issue with downgrades has been rapidly recognized by the medical community, which has been requesting a DF-4 to IS-1 adapter, which none of the manufacturers has been willing to develop and which is unlikely to ever be available. Although workarounds have been reported in certain patients with CRT-D and IS-1 LV leads,^5^ most patients will wind up with an additional IS-1 lead and a redundant DF-4 ICD lead.

#### Conduction system pacing

**Figure 1:**
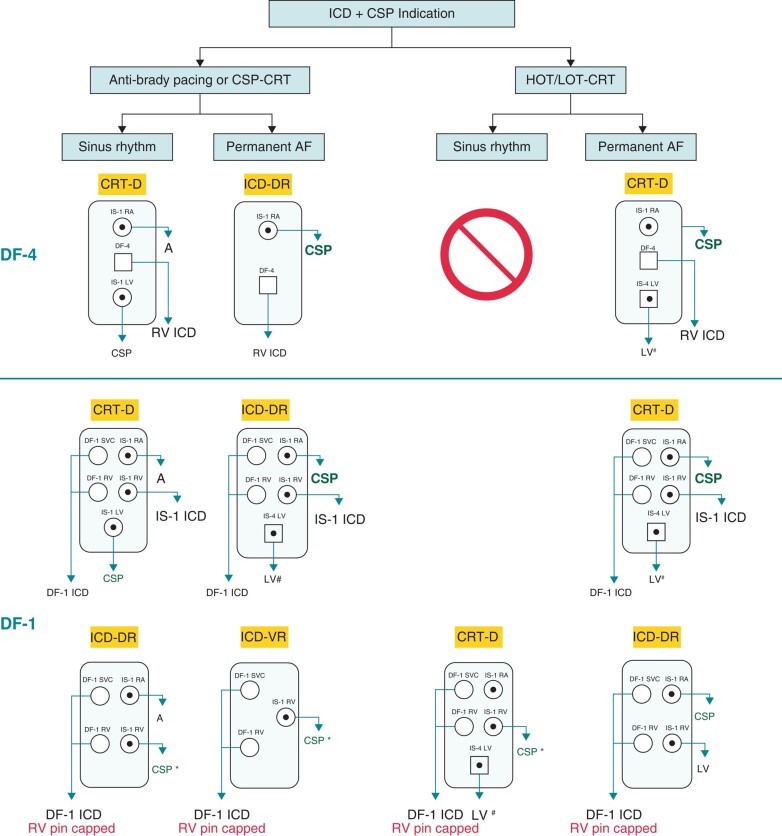
Different configurations for delivering conduction system pacing in defibrillator patients.^#^IS-1 LV lead may also be used; *if His bundle pacing is used, good sensing must be demonstrated with adequate R-wave amplitude and absence of atrial/His oversensing.

Recently, conduction system pacing (CSP) is emerging as a popular ventricular pacing site in patients requiring a high percentage of ventricular pacing, be it for atrioventricular conduction disease or for CRT.^6^ This technique provides an appealing option for many ICD patients and may be delivered by connecting the CSP lead to the left ventricular IS-1 port of a CRT-D, or to the atrial port of a dual-chamber ICD in patients with permanent atrial fibrillation. In patients with a DF-1 ICD, the CSP lead can be connected to the right ventricular IS-1 port of the generator *in lieu* of IS-1 part of the ICD lead, which is capped (*Figure 1*). This configuration has been used to deliver an economical form of CRT and has been shown to be safe and effective.^7^ It is of particular interest in an ICD patient who develops an anti-bradycardia pacing or a CRT indication over follow-up, and who requires an upgrade to physiological pacing. Unfortunately, the DF-4 standard does not allow the addition of a pace–sense lead. As we currently have no ICD lead capable of providing CSP, the only option will be to replace the generator with one that has an additional IS-1 port (even if the DF-4 generator has significant residual battery longevity). The only disadvantage is that the system is rendered non-MRI-conditionally safe.^8^ Nevertheless, there is consensus that MRIs may still be safely performed even in the presence of an abandoned lead, if this is deemed necessary.^9^

An additional scenario is combination of CSP with pacing of the left ventricle via a coronary sinus lead (HOT-CRT or LOT-CRT), which is a strategy intended to maximize electrical resynchronization in selected patients in whom CSP or biventricular pacing yields suboptimal results.^10^ In CRT-D patients who are in sinus rhythm (i.e. who also require an atrial lead), the only option is to use a DF-1 ICD and to connect the CSP lead to the right ventricular IS-1 port—see *Figure 1*.^6^

#### Ventricular sensing or pacing issues

Defibrillator algorithms rely heavily on an appropriate ventricular sensed signal to account for the different vectors during sinus rhythm as well as during ventricular tachyarrhythmias. In some patients, the ventricular sensed signal decays or the capture threshold increases over time, for example due to calcification of the lead tip. With a DF-1 connector, the addition of a separate pace–sense lead with abandonment of the IS-1 part of the ICD lead can overcome this problem.^11^ This avoids the extraction of the old defibrillator lead as well as the addition of a second ICD lead.

A similar situation arises if the low-voltage component of the ICD lead shows evidence of a fracture. In most of these instances, it is probably advisable to revise the lead as the fracture may later progress to also involve the high-voltage components,^12^ but there may be cases where it is preferable to simply add a pacing lead to ensure ventricular sensing and/or capture and avoid inappropriate shocks.

Yet another (currently rare) situation is a functioning ICD lead that is under recall in a CRT-D patient with an IS-1 left ventricular lead. In this instance, the right and left ventricular leads may be switched to avoid risk of inappropriate shocks in case of ICD lead fracture.^13^

### Clinical scenario for the need of an additional implantable cardioverter defibrillator coil

#### High defibrillation thresholds

Defibrillation threshold testing is seldom performed now and most patients receive single-coil ICD leads. Some patients may have high defibrillation thresholds that are not diagnosed at implantation, with a ‘near-miss’ of sudden death following failed appropriate ICD shocks. In these patients, an option to reduce defibrillation thresholds is to upgrade the system with a dual-coil system. Additional ICD coils are available (e.g. Medtronic Transvene 6937A) that may be implanted in the superior vena cava, or in the coronary sinus or azygos vein (with possibly greater efficacy of the shock vector in these alternative sites) and high-voltage splitter connectors allow for multiple configurations for shock delivery. These strategies are only possible with a DF-1 system, which avoids having to add a new ICD lead with extraction/abandon of the single-coil DF-4 lead.

### Too many industry standards

Currently, there are four lead connector standards available: IS-1 (for atrial, right ventricular, or bipolar left ventricular leads), IS-4 (quadripolar left ventricular leads), DF-1 (high-voltage port of ICD lead), and DF-4 (combination of high-voltage and low-voltage components of the ICD lead). This means that instead of the original three ICD models with the IS-1/DF-1 standard (VR, DR, and CRT-D), there are now eight. For CRT-Ds, there are four possible devices: IS-1/DF-1, IS-4/DF-1, IS-1/DF-4, and IS-4/DF-4. These different permutations and combinations have made it complicated to manage production and device stocks and also create confusion at implantation (instances of the wrong generator being unpacked are not exceptional). Abbott, Boston Scientific and Microport are not for the time being equipping their new ICD platforms with the DF-1 standard, while Biotronik and Medtronic are continuing to do so. We strongly believe, however, that given the many possible advantages of the DF-1 standard, both, the DF-4 and DF-1 standards, should both remain available on the market.

## Conclusion

Although the DF-4 defibrillator standard is convenient during implantation and has been proved to be safe and has therefore become the preferred system for many ICD implanters, it precludes flexible therapeutic options during follow-up. The main issues are that DF-4 systems do not allow downgrading to a pacemaker nor do they offer the possibility to upgrade to CSP without using a new ICD-generator. The addition of pacing leads for sensing/pacing issues of the ICD lead or of standalone ICD coils/subcutaneous arrays for high defibrillation thresholds are rarer situations but are at least options that are available with the DF-1 standard to handle these complex situations, should they arise.

The uptake of conduction system pacing is swaying practice towards increased use of DF-1 ICDs, which is of interest in case a pacing indication arises over follow-up. It also offers an economical solution for resynchronization therapy in low-income countries, where this treatment may be afforded by more patients compared to premium CRT-D device. The few advantages of DF-4 ICD leads have been a high price to pay for giving up the options and flexibility offered by DF-1 systems, and hopefully market forces will drive industry partners to keep offering this standard for the benefit of our patients.

## Data Availability

All relevant data are within the manuscript.
